# Ex Vivo and In Vivo Stem Cells-Based Tissue Engineering Strategies for Their Use in Regenerative Medicine

**DOI:** 10.1155/2018/7143930

**Published:** 2018-04-15

**Authors:** Víctor Carriel, Stefano Geuna, Miguel Alaminos

**Affiliations:** ^1^Department of Histology, Tissue Engineering Group, University of Granada and Instituto de Investigación Biosanitaria, Ibs, Granada, Spain; ^2^Dipartimento di Scienze Cliniche e Biologiche, Università di Torino, Torino, Italy

Tissue engineering is an emergent discipline focused on the generation of bioartificial tissue-like substitutes through the combination of biomaterials, cells, and growth factors. In this context, stem cell-based strategies have made substantial contributions in the field. Researchers worldwide have demonstrated that stem cells have an extraordinary capability to differentiate into different cell lineages, promote tissue regeneration, release a wide range of growth factors, and modulate the host immune response. Based on the current impact of tissue engineering and stem cells in medicine, *Stem Cells International* set out to publish a special issue focused on the “Ex Vivo and In Vivo Stem Cell-Based Tissue Engineering Strategies for Their Use in Regenerative Medicine.” This special issue resulted in a collection of nine outstanding articles which include two reviews and seven original studies made by recognized researchers from seven countries across Europe, Asia, South America, and North America.

Concerning the review articles, M. Cimino et al. contributed with a complete review entitled “Xeno-Free Strategies for Safe Human Mesenchymal Stem/Stromal Cell Expansion: Supplements and Coatings,” where authors suggest that culture/expansion of human mesenchymal stem cells under xeno-free conditions is still needed to improve their clinical translation. The review made by A. Owczarczyk-Saczonek et al. discussed “The Use of Adipose-Derived Stem Cells in Selected Skin Diseases (Vitiligo, Alopecia, and Nonhealing Wounds),” highlighting that these stem cells are promising alternatives to generate new engineered or stem cell-based strategies to treat skin diseases.

In the field of cartilage tissue engineering, two interesting *in vivo* studies were published in this special issue. On the one hand, M. Mata et al. published the article entitled “In Vivo Articular Cartilage Regeneration Using Human Dental Pulp Stem Cells Cultured in an Alginate Scaffold: A Preliminary Study” in which they demonstrated, through different histological approaches, that human dental pulp stem cells have a positive impact on the regeneration of articular cartilage in rabbits. On the other hand, V. Chapman et al. demonstrated that late passage marrow-derived mesenchymal stem cells were more efficient than early passage cells in the treatment of osteoarthritis on their article entitled “Therapeutic Benefit for Late, but Not Early, Passage Mesenchymal Stem Cells on Pain Behaviour in an Animal Model of Osteoarthritis.” These articles provide new evidence related to the usefulness of stem cells in cartilage tissue engineering.

Currently, adipose-derived mesenchymal stem cells are considered one of the most promising mesenchymal stem cells by several and well-supported reasons. Within this special issue, N. Garcia-Honduvilla et al. demonstrated that a natural extract purified from *Cryptomphalus aspersa's* eggs induced the differentiation of adipose stem cells to myofibroblast on their study entitled “High Sensitivity of Human Adipose Stem Cells to Differentiate into Myofibroblasts in the Presence of C. aspersa Egg Extract.” In another interesting ex vivo approach, E. Oliveira et al. on their article “Influence of Different ECM-Like Hydrogels on Neurite Outgrowth Induced by Adipose Tissue-Derived Stem Cells” demonstrated the synergetic effect of the correct combination of biomaterials and adipose stem cells to increase the neurite outgrowth from DRG explants. In these articles, the usefulness and versatility of adipose stem cells in tissue engineering were well demonstrated.

Extraembryonic tissues are an important source of stem cells and natural scaffolds. In this regard, G. P. Liao et al. successfully repaired diaphragmatic defects in rats by using decellularized rat diaphragm containing human amniotic fluid-derived stem cells on their article entitled “Tissue Engineering to Repair Diaphragmatic Defect in a Rat Model.” In addition, in order to solve the problem associated to the expansion of epithelial cells, S. M. Nam et al. investigated the use two stem cells sources as feeder cells of corneal epithelial cells on their article entitled “Ex Vivo Expansion of Human Limbal Epithelial Cells Using Human Placenta-Derived and Umbilical Cord-Derived Mesenchymal Stem Cells.” In an *in vivo* approach, M. Garrido et al. demonstrate the potential clinical application of amniotic membrane in digestive surgery on their article “Transplantation of Human Amniotic Membrane over the Liver Surface Reduces Hepatic Fibrosis in a Cholestatic Model in Young Rats.” These three articles highlight the potential clinical usefulness of extraembryonic-derived cells and scaffolds in tissue engineering.

Finally, in these nine articles, authors rigorously discussed and demonstrated the high versatility of different kinds of stem cells to differentiate, promote tissue healing, modulate host immune response, and serve as feeder platform for the ex vivo expansion of differentiated cells. In conclusion, the articles published in this special issue provide new tissue engineered-based strategies and scientific evidence that support the potential clinical usefulness of stem cells in regenerative medicine.

*Víctor Carriel* *Stefano Geuna* *Miguel Alaminos*

